# Economic benefits of high value medicinal plants to Pakistani communities: an analysis of current practice and potential

**DOI:** 10.1186/1746-4269-10-71

**Published:** 2014-10-10

**Authors:** Hassan Sher, Ali Aldosari, Ahmad Ali, Hugo J de Boer

**Affiliations:** Center for Plant Sciences and Biodiversity, University of Swat, Saidu Sharif, Pakistan; Department of Geography, College of Arts, King Saud University, Riyadh, Kingdom of Saudi Arabia; Department of Organismal Biology, Uppsala University, Uppsala, Sweden; Naturalis Biodiversity Center, Leiden, The Netherlands; Natural History Museum, University of Oslo, Oslo, Norway

**Keywords:** Economic development, Market ethnobotany, Medicinal plants, Tribal communities, Value chain analysis

## Abstract

**Background:**

Poverty is pervasive in the Swat Valley, Pakistan. Most of the people survive by farming small landholdings. Many earn additional income by collecting and selling plant material for use in herbal medicine. This material is collected from wild populations but the people involved have little appreciation of the potential value of the plant material they collect and the long term impact their collecting has on local plant populations.

**Methods:**

In 2012, existing practices in collecting and trading high value minor crops from Swat District, Pakistan, were analyzed. The focus of the study was on the collection pattern of medicinal plants as an economic activity within Swat District and the likely destinations of these products in national or international markets. Local collectors/farmers and dealers were surveyed about their collection efforts, quantities collected, prices received, and resulting incomes. Herbal markets in major cities of Pakistan were surveyed for current market trends, domestic sources of supply, imports and exports of herbal material, price patterns, and market product-quality requirements.

**Results:**

It was observed that wild collection is almost the only source of medicinal plant raw material in the country, with virtually no cultivation. Gathering is mostly done by women and children of nomadic Middle Hill tribes who earn supplementary income through this activity, with the plants then brought into the market by collectors who are usually local farmers. The individuals involved in gathering and collecting are largely untrained regarding the pre-harvest and post-harvest treatment of collected material. Most of the collected material is sold to local middlemen. After that, the trade pattern is complex and heterogeneous, involving many players.

**Conclusions:**

Pakistan exports of high value plants generate over US$10.5 million annually in 2012, with a substantial percentage of the supply coming from Swat District, but its market share has been declining. Reasons for the decline were identified as unreliable and often poor quality of the material supplied, length of the supply chain, and poor marketing strategies. These problems can be addressed by improving the knowledge of those at the start of the supply chain, improving linkages among all steps in the chain, and developing sustainable harvesting practices.

## Background

The phrase “High Value Minor Crops” refers to plants that are relatively small contributors to a country’s agricultural output. They fall into two major groups: herbs and spices; and medicinal and aromatic plants (MAPs). Although individually small contributors to output, the importance of these plants in total is evident from the fact that in 2006, their global trade reached US$ 60 billion [1, 2]. Europe alone annually imports about US$ 1 billion in MAPs from Africa and Asia [3, 4]. Such trade is expected to expand substantially by the year 2050 [5] because of the increasing popularity of herbal medicines [6, 7].

The total contribution to national agriculture output of MAPs is small, but their value per weight is among the highest among traded plants. The pharmaceutical cash crops have a huge potential for remote communities that practice subsistence agriculture and have limited access to regional economy [8, 9]. In many parts of south and western Asia MAPs have been collected or cultivated and traded for centuries [6, 10–14].

In Swat District in Khyber Pakhtunkhwa Province of Pakistan, the present supply of MAPs comes almost entirely from wild-harvested material, and not from cultivation. A large number of rural households in Swat District collect MAPs, at the very least as informal gatherers for local use [4]. Many rural households that market MAPs gather these from forests and fields, and are nomadic tribesman and themselves are small and marginal farmers. Any MAP cultivation usually constitutes only a small part of the household’s farming operation.

Earlier studies have estimated that during the spring and summer MAP collection and trade becomes the main source of household income for 5,000 or more traditional nomadic tribesmen who gatherer these plants from the wild in Swat District [4, 7, 15]. The majority of nomadic gatherers and small-farm collectors have little or no professional training. The collection is seldom systematic and controlled, and as a result wild herbals have come into disrepute on account of the haphazard collection, non-grading and improper care in drying and storage. Sometimes adulteration with spurious plants is practiced with the motive of quick financial gain. However, because many of the nomadic tribesman and farmers in the mountains of the Swat District live near subsistence level, and collection, and potentially cultivation, of MAPs could gain more importance as a source of supplementary income. Most of the households in Swat District, including those collecting MAPs, are still living in poverty [4, 7]. At the same time, many collected plant species have a high market value, but the collectors usually do not know how to market them [15].

To enhance incomes from MAPs, collectors require a better understanding of the needs of individual markets. This is critically important, especially regarding quality specifications and their implications for pre and post-harvest management and proper product handling. The study for Swat District was, therefore, initiated to identify constraints such as a fragmented information base, training and educational deficiencies, an uncoordinated approach to collection and marketing of MAPs species, and the need to clearly identify traders and markets.

The overall objective of this study was to examine how to increase the value and expand the benefit of MAPs collection, cultivation, processing and marketing for people in Swat District. The specific objectives were to attain sustainable harvesting practices, small scale cash crop cultivation and local processing of raw material to add value before marketing. In the study we look at the various steps in the supply chain, from collector/farmer to final domestic market or exporter, examining the ways in which the market value of both raw and processed MAPs could be improved. For Pakistan, economic analysis of the marketing chain of MAPs, from collection to consumption, has been limited.

This paper concentrates specifically on describing Swat District and the procedures and results from surveys and focus group meetings undertaken to assess the amount of collections, marketing channels, and prices at each level for the MAPs originating in Swat District. This study was launched in June 2012 to respond to the above challenges and to support the 2011 Framework for Economic Growth [16].

## Methodology

### Study area

**Figure 1:**
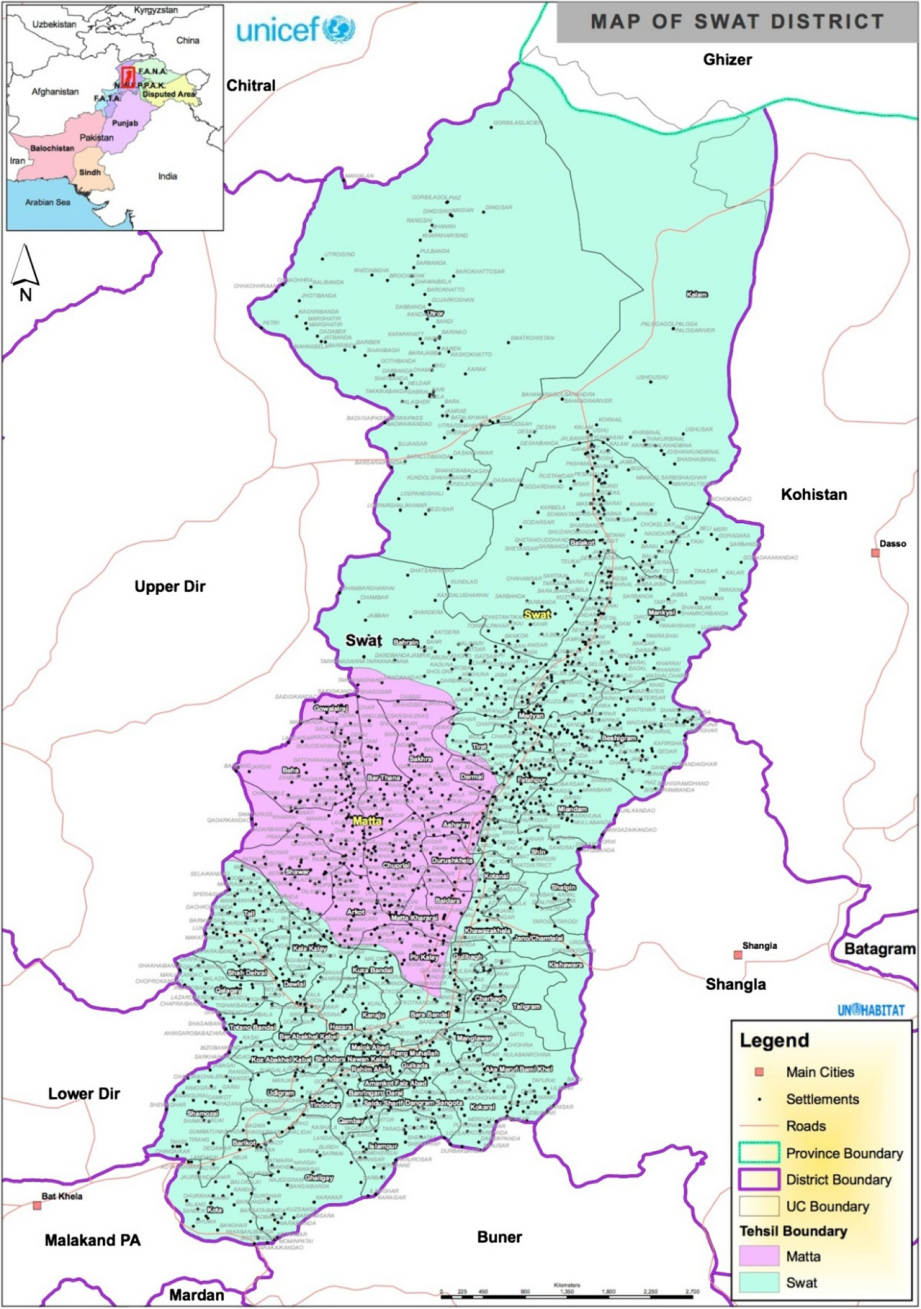
**Map of Swat District (Source: UN Habitat, Forestry Department, Mingora, Swat).**

Swat District is part of Malakand Division of Khyber Pakhtunkhwa Province and lies in northwestern Pakistan (Figure 1). The district is bordered by Chitral in the north-west, Gilgit in the north-east, Shanglapar in the east, Buner in the south, Dir in the west, and Malakand Agency in the south-west. Swat District has an area of is 5 337 km^2^ and has a population of about 1.3 million with a density of 230/km^2^, and annual population growth rate of 3.48 percent [17].

Swat District is very mountainous, and its highest peak, Falaksair, measures 6,098 meters in height. The district occupies the floristically rich southern extension of Hindu Kush Raj of the Hindu Kush mountain range. It was originally known as Uddiyana (Garden) during its Buddhist bloom in the 8^th^ century AD. The Buddhists admired it for its peace and beauty, it was the envy of the Mughals [18]. The main ethnic groups in the district today are Yousafzai Pathans, Kohistani and Gujars. The Yousafzai Pathans are the descendants of Ghazni Afghans. The Gujars and Kohistanis, who speak different dialects of Gujri, Garwi, Torwali, and Kohistanis, inhabit the upper mountainous areas of the district.

The area is not climatically uniform, and altitude and exposure greatly modify the climatic conditions within the area. On the basis of altitude, climate and vegetation, the area can be divided into many climatic and vegetation zones that vary from sub-tropical chirr pine forest to alpine pastures and meadows. Similarly, the diversity of climate and topography of Swat District is reflected by the three main phytogeographic regions: 1) Sino-Japanese region, 2) Irano-Turanian in the north-north west and 3) Saharo-Sindian region in the south [19].

Several species of commercially important medicinal plants are collected from temperate forests, sub-alpine forests and alpine meadows. Many of these species are slow growing perennials that require a number years of vegetative growth before flowering. Due to overgrazing, unsustainable collection and illicit cutting of the forests, most of these species are now classified as endangered. If present rates of unsustainable collection and other inimical factors continue most of the high value potential MAPs will become locally extirpated from many parts of Swat District [18].

### Economic features of Swat District

Swat District has a generally narrow economic base characterized by agro-pastoralism. The potential for diversifying the economy is rather limited due to remoteness and inaccessibility, the harsh climate, lack of managerial and technical skills, lack of financial resources, lack of physical infrastructure, high cost of doing business in the area, low level of demand for different economic goods and services, and high cost of production in the area [20, 21].

Nevetheless, Swat District can develop through increased forestry and agriculture production in general and through capacity building of medicinal and aromatic plant collectors/farmers in particular. Capacity building training can improve skills of MAP collectors and cultivators, which will enable them to fetch better prices for their quality produce. This sector, can, therefore, be developed mainly through cultivation and intensification to meet domestic requirements.

### Selection criteria for Swat sub-valleys

Collection and marketing of MAPs was evaluated in this study for six Swat sub-valleys: Miandam, Madyan, Behrain, Kalam, Lalku, and Sulatand of Swat District. These sub-valleys were selected partly based on earlier studies of HS [18] and partly in view of information obtained early in the study during the focus group meetings with the local communities that are described below. The seasonal extremes in environmental conditions make the local diversity of plants vary in parallel to the regional diversity. A short but intensive growing season results in a temporal flush of plant species diversity depending on elevation and other aspects. Local and regional diversity are enhanced by the variety of microclimates and environmental conditions. The selected sub-valleys represent the alpine meadow, sub-alpine pasture, alpine scrub, moist, dry temperate coniferous forest and oak scrub zones of the outer Hindukush Mountain Range. This representation of sub-valleys was considered to reflect the presence of the species of concern in other valleys of Swat District as well.

### Urban markets for MAPs

Three cities are the main final markets for traded MAPs once they are moved out of Swat District, Peshawar, Lahore and Karachi. **Peshawar:** The MAPs sold in Peshawar herbal markets are generally obtained from Swat District, Lahore, and Afghanistan. Peshawar markets also supply a number of imported MAPs to Swat District and Afghanistan for local uses. The market receives large quantities of herbal materials from Swat District that are subsequently supplied to Lahore. **Lahore:** Most of the dealers in Lahore herbal market are trading crude herbs imported from India, directly or indirectly. Over 50% of materials traded in Lahore are of Indian origin, and this is mainly due to cross border trade via train. The Lahore herbal market acts as a hub of national trade of MAPs. It is not only catering to the needs of smaller markets in various cities and towns of the province of Punjab but also supplies considerable quantities of materials to the Karachi market. The middlemen of the MAPs trade usually bring the materials from Swat District to Lahore. **Karachi:** Most of the crude herbal items traded in Karachi markets are obtained from the Lahore herbal markets. However, a few agents also bring the material directly from up-country, including Swat District. Prices of various items in Karachi market are generally 10-20% higher than Lahore, reflecting higher transportation, higher labor costs, and profits of additional middlemen. Both the Lahore and Karachi herbals markets are the major suppliers of materials to the large national herbal pharmaceutical companies. These companies generally purchase materials through middlemen or so-called suppliers.

### Study procedures

The market study of collection and trade patterns for high value MAP species was conducted during summer 2012 with particular reference to plants from Swat District. Interviews with MAPs collectors/farmers, local dealers and hakims (*wise man* in Arabic, and in Pakistan referring to a person who has expertise in diagnosing diseases and treats them with the use of formulations containing mainly MAPs) were conducted at different potential production sites of the district. Interviews were also conducted at markets in regional and major cities of Pakistan, specifically Mingora, Peshawar, Lahore and Karachi. Information was gathered as to how and from whom the plants were obtained and to whom they were sold [22]. Likewise shopkeepers were asked about the sources of the MAPs received by them. Respondents were also asked about the total quantity of each species collected from the wild, and these were then summed to represent the total of the six sub-valleys. This information was documented during surveys of collectors, traders, and also in focus group discussions.

Different herbal pharmaceutical companies were visited, and information on the national and international trade of MAPs compiled by the Export Promotion Bureau (EPB) and Forest Department was consulted. Technical literature relating to collection, trading, processing and national and international marketing of MAPs was also consulted. Additionally, the dealers and suppliers catalogues and brochures of companies involved in the trade of MAPs were reviewed during this study. Accordingly, the areas surveyed can be broadly classified as *sources* and *markets.*

- Interviews with collectors, dealers and other knowledgeable persons about the local use and trade provided information on the sources of MAPs.
- Interviews with local, regional and national traders, EPB, and interviews with hakims and representatives of herbal pharmaceutical companies provided information on the markets for MAPs.

Two different questionnaires, one for collectors/farmers and another for dealers/pharmaceutical companies, were used to conduct this production and marketing value-chain analysis. After preliminary work, the questionnaires were modified to improve the correspondence of the surveys with the objectives of the study.

Voucher specimens were collected of all reported species and identified using existing floras and floristic accounts for the region [19, 23, 24]. Nomenclature follows the accepted names and authors in The Plan List [25]. Botanists from the National Herbarium, National Agricultural Research Council (NARC), Islamabad confirmed the identifications, and the collected voucher specimens were deposited at the National Herbarium.

### Survey of collectors

In studying Swat District as a source, we examined the collection patterns of MAPs as an economic activity and the likely destinations of these products in national or international markets. Information and data on various aspects of collection, such as collection method, time, and marketing of each species were attained from local collectors and farmers through surveys and interview discussions. For this purpose collectors, farmers and traders of ages 20–60 years were surveyed using a questionnaire to document their knowledge of MAPs. Information was gathered as to how, and from where the respondents collect MAPs and to whom they sell. Respondents were also asked about their annual income earned from the sale of targeted plants and returns to the work invested. Surveys were conducted with a total of 120 collectors and farmers participating in the sale of MAPs in various collection and cultivation sites in Swat District. Collectors were targeted by identifying those who had received some training in collection and procession of MAPs by NGOs in the region and using a snowball identification process and focus group information. The surveyed collectors and farmers are the main group bringing MAPs gathered in the forests and fields into the market. However, we do not have a precise estimate of what proportion of the MAPs brought to market in total are handled by the surveyed set of collectors.

### Focus group discussions in three villages

Focus group discussions (FGDs) were conducted in a local Hujra (meeting place) in three villages. The participants in the FGDs were village elders, MAPs collectors/farmers, local traders, representatives from Non Timber Forest Products Directorate and from the Forest and Agriculture Departments. During FGDs the objectives of the study were described to the participants. The purpose of the FGDs was to focus on specific issues/topics and possible options for activities like data collection on MAPs marketing and production, and cultivation of selected commercially important medicinal plants on the farm land of community members participating in the FGD. Participation was encouraged by all and efforts made to ensure that no one dominated the discussion. Records were kept of the discussions and observations of each FGD, and generally each FGD lasted for 2–3 hours. The FGDs were designed in a manner to be interactive and participatory and were facilitated by the author of this paper. About 350 MAPs collectors and farmers from the three villages participated in the FGDs. These meetings were a very important aspect of the study as they helped in the identification of MAPs collectors, farmers, local traders, wholesalers, primary and secondary middlemen, and MAPs exporters and the important issues they faced.

### Survey of traders, hakims and herbal pharmaceutical companies

In studying the market chain of MAPs, we also identified and interviewed local, regional and national traders, hakims and representatives of pharmaceutical companies. This served as another important source of information on various aspects of MAPs trade from Swat District in particular and the country in general. Information was collected about major items for sale, source and origin of crude MAPs, approximate quantities of MAPs material transacted annually, annual income of traders, and common problems in this business. The standards and product-quality requirements of traders from collectors/farmers and local dealers for domestic and export-market formulations were also noted. Interviews with 50 participants involved in the trade and business of MAPs were conducted, in addition to the 120 surveys of collectors/farmers mentioned above.

## Results

Value-added activities currently carried out by the collectors and wholesale and retail dealers in the area include product cleaning, drying, cutting, and, in some cases, washing of the plants or plant parts to be sold. However, these activities are usually conducted only to the extent needed to meet the minimum quality standard required by the local market [26] and without regard to modern management techniques. This suggests that collectors and dealers would benefit from training in how to better meet market needs and education on how adding value can impact demand and sales revenue.

In Pakistan in general and in Swat District in particular a number of organizations in the public and private sectors are undertaking research and development work on MAPs. However, the systematic documentation of MAPs regarding production and marketing is not carried out by any public or private organization. Information availability has largely been in an ad hoc and uncoordinated manner in all sectors from production to trade, due to absence of consensus on strategic thrust areas. However, sustainability of MAPs production and trade will depend on the principle that it meets the need of the present without compromising the ability of future generations to meet their needs.

Final MAPs markets are split primarily into three segments: domestic commercial; export; and local hakims, other traditional healers and retail shops. The major domestic manufacturers, like Hamdard, Qarshi, Ajmal and others, produce 300–400 medicinal products. However, collectors do not typically have linkages with these markets, and, therefore, must rely on local traders to sell their products within the existing value chain. Collectors are, therefore, often isolated from the final consumers of MAPs products, and do not have a good understanding of market needs beyond the limited information provided by the traders.

Interviews conducted in this study indicate that the trade of MAPs species began in Swat District about two decades ago. Individuals from India and Karachi visited Mingora and Madyan to obtain plants for both domestic markets and international trade. Further development occurred when Sandoz Pharmaceuticals launched ex-situ cultivation of three species in the area, *Sinopodophyllum hexandrum* (Royle) T.S. Ying (syn. *Podophyllum hexandrum* Royle)*, Saussurea costus* (Falc.) Lipsch. (syn. *Saussurea lappa* (Decne.) Sch.Bip.) and *Viola pilosa* Blume (syn. *Viola serpens* Wall. ex Ging.), with the aim of augmenting local gathering of these species from the wild. The business of these herbal materials was quickly picked up by some people in Mingora, Madyan and Behrain and is now well established. Mingora and the smaller city of Madyan have become the main supply centers of herbal material from Swat District for the national and international markets. The cultivated plants are mainly used by the pharmaceutical companies.

The study found that during the spring and summer of 2012 collectors and farmers marketed 80 MAPs for commercial use. Most of the harvesting activity took place in the summer but three species (*Morchella esculenta* Fr.*, V. pilosa* and *Colchicum luteum* Baker) were gathered from March to May. Most of the species were sold to wholesalers via middlemen. Of the 80 species, 24 were high value MAPs collected in relatively large amounts for sale. These are eventually sold either in national markets outside Swat District or international markets. Some quantities of these species, and largely the remaining 56 MAP species, are collected and traded by hakims in their medical clinics and in shops at local herbal markets. Hakims mostly collected these plants themselves or had them gathered by adult collectors or children who they rewarded by treating them as patients free of charge. In this way the hakims make a good profit from the sale of MAPs. However, the informal collectors expressed satisfaction with this arrangement. They reported typically gathering 2–3 maunds (1 maund=40 kg) of plant materials per year on the demand of hakims.

The study found that the MAPs species were mainly collected from the forest areas of Swat District and the collectors sell the material fresh or semi dried. Collection of plant material in Swat District appears to be on a “first-come, first-served” basis. There is no coordinated management structure involved. Likewise, the government exercises little control on collections, although there is a permit system that is intended to regulate and assess taxes on the gathering of the roots of these species.

Another problem identified in the market chain interviews, one that will probably become more severe in the future, is that many of the MAPs currently being collected in Swat District are now being produced and exported from other countries, including India and China. In 2012, plant material from these areas was competitive in price and often of better quality. The difference in quality resulted in buyers showing less interest in plant material from Swat and/or offering lower prices for it. During the interviews in Karachi it was frequently mentioned that the material of Swat District is traded in the Karachi market as imported material (without mentioning its origin from Swat District) to make it more acceptable to the buyers.

### Chain of commercialization

**Figure 2:**
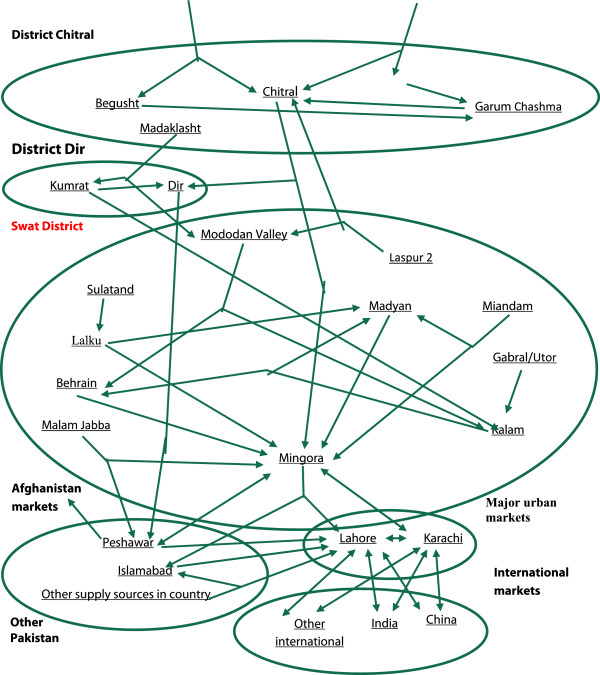
**Supply routes from MAP collection sites and other areas to local, national and international markets.**

Figure 2 displays the marketing channels identified in the interviews of MAPs market participants. The study shows that Mingora is the main trade center for many high value plants in Swat District. Mingora supplies considerable quantities of plants to various national trading centers in Pakistan, including Peshawar, Lahore and Karachi. The direct linkages in the market channels between the various herbal markets in Swat District and the national and international levels are shown in Figure 2. Mingora receives material from various hilly areas, while Lahore herbal market acts as the major center of trade in the country receiving imported material from abroad and from the country sources. Karachi is a key export terminal.

In Swat District, the collection and trade of high value MAPs is highly uncoordinated and varying (i.e. from area to area and species to species). One common marketing channel for obtaining plants from Swat District is that dealers from the national market send representatives to local dealers (beopari) in Mingora to put up their demand. The local dealers pass the message to their agents, mainly local shopkeepers in the valleys. These agents inform small shopkeepers and collectors. The collectors bring the species gathered themselves and other tribesmen to the local shopkeepers and agents. Collectors sell the MAPs at a small margin higher than they pay to the gatherers, and most gatherers and collectors are illiterate and do not negotiate higher prices for the plant materials. As a result, they gather large and increasing quantities without receive high returns. The dealers of Mingora get the material from the agents when it is ready, and in this way the plant materials passes through three or four middlemen before wholesale.MAPs are traded in a wide range of materials that are used in medicine and health products in various forms or products that contain elements of these plants. In the regional market of Mingora materials are sold to wholesalers directly or through the middlemen generally known as commission agents. These materials are transported to the wholesalers in the bigger city markets and from there the materials are either stored for export, sold to retailers, or supplied to the manufacturers. In some cases, the specific demand comes from wholesaler dealers who recruit their agents to organize the collection of the required materials, as described above. However, this study found that the interplay of middlemen in trade often acts as a shield, blocking communication between the primary collectors of the MAPs and their consuming centers. The limited access to markets and dependence on intermediaries has a direct effect on the prices. The study concludes that approximately 320 large wholesalers are operating in the markets located in Mingora of Swat District and in main cities of the country. Additionally, markets are located in some smaller towns of Swat District like Madyan, Miandam and Behrain (see Figures 1 and 2), as they are in close proximity to MAPs growing areas.

Pakistan is involved in both domestic production, trade and consumption of MAPs and also in international trade [27]. However, only limited economic analysis of MAPs exists for marketing chains from collection to domestic consumption or export. All available data relates to quantities traded in markets at a specific time and their approximate value. Mostly traders were reluctant to disclose volumes of trade. However, it is known that trade in MAPs is dominated by wholesaler dealers. At the retail level, small shopkeepers, pansar stores, and some hakims rely on wholesalers for their supply or operate in a more informal manner.

The marketing of MAPs faces diverse problems encountered at various stages. The raw MAPs are either sold dried or fresh to the local commission agents and shopkeepers who sell them to wholesalers. The wholesalers sell them to the pharmaceutical companies or to exporters. The collection and trade of crude MAPs is very erratic in many parts of the country including Swat District. Quantities collected in the wild are always uncertain. Thus, the availability of particular crude MAPs remains very unstable and market trends cannot be easily determined. Prices fluctuate greatly due to variation in external and internal demand within the country. The prices also fluctuate greatly due to variation in rates even in the same market, and it is difficult to ascertain actual rates of particular MAP species. The margin of profit earned by the traders arises as they purchase the crude MAPs at nominal rates from collectors and producers and obtain higher prices as the products move up the marketing chain. This study revealed that market information is primarily price oriented but has flaws because the reported prices do not show how product quality, volume traded, consignments size, or origins affect the price. The prices are disseminated without analysis.

### Constraints and opportunities in MAP collection and marketing

**Figure 3:**
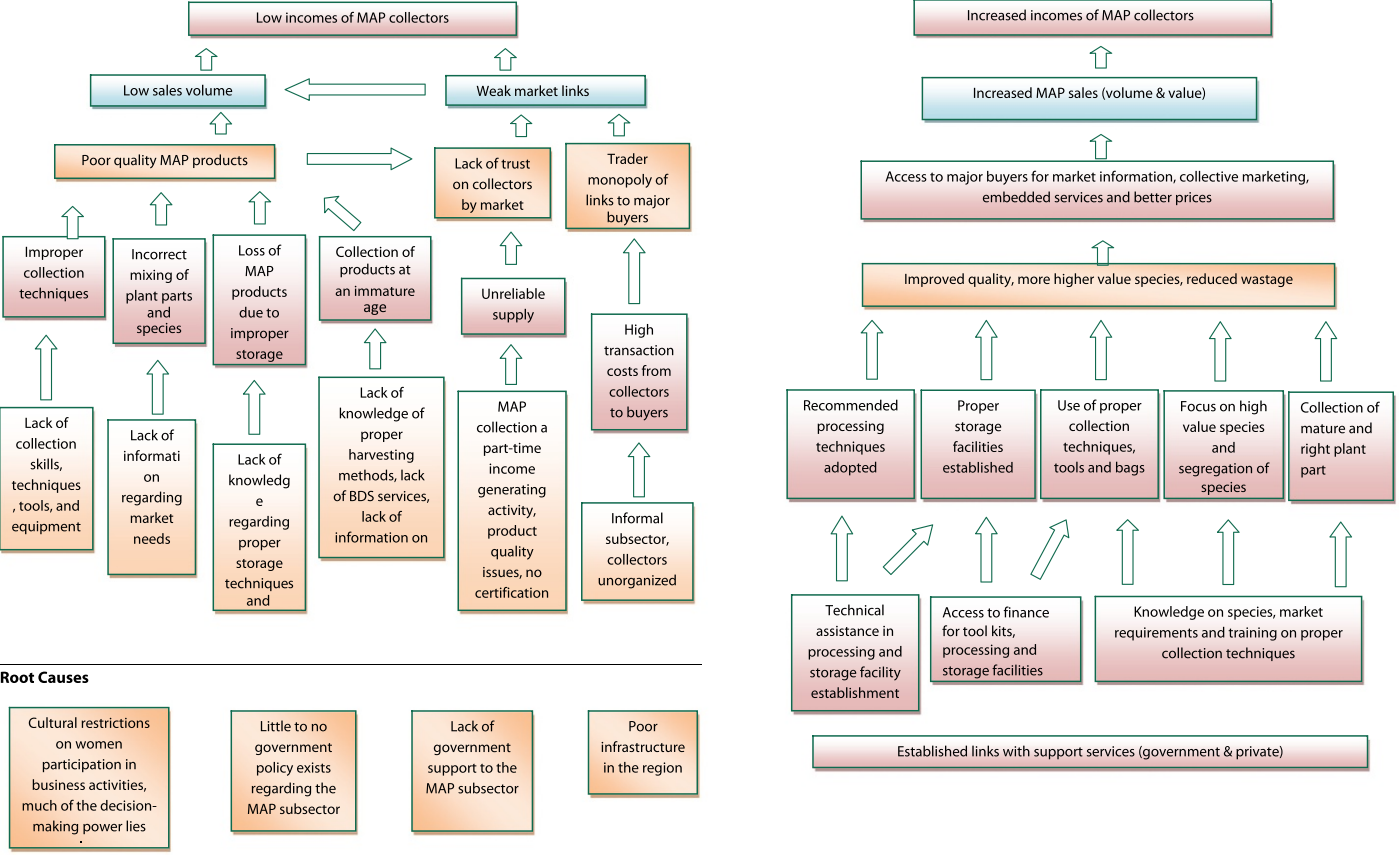
**Problem tree for MAP value chain.**

Figure 3 provides information regarding the problems, constraints and solutions within the MAP marketing chain, as compiled and synthesized based on the interviews and focus group discussions. The study documented that MAPs trade is highly complex, uncoordinated and heterogeneous, involving many players. The study showed that approximately 90% of MAPs materials are sold unprocessed by collectors. Local collectors are untrained in sustainable harvest methods, post-harvest handling, and the proper storing of medicinal plants. They have weak market links and are unable to negotiate the prices of collected MAPs. Many collectors have small volumes/quantities of MAPs and so receive only a small portion of the total revenue.

The current value-added practices carried out locally in the region include cleaning, drying, cutting, and in some cases washing of the plants (or plant parts). However, these practices are often lacking at the collectors/farmers level, and these practices are usually done with the purpose of meeting only the minimum quality standard required in the national market. Plants were often dried in humid, dark areas where they are subject to infestation by microorganisms and consumption by insects. Therefore, collectors get minimum monetary return for their MAPs, and products from Swat District face the quality concerns noted above. Secondly, lack of knowledge about the part used and time of collection has led to misuse of the species. Appropriate timing of collection of desired plant parts during the year and lifescycle can have a major impact on the yield percentage and quality of therapeutically active ingredients.

In addition, buyers further down the value chain often dictate terms to local traders who buy directly from collectors, including the size of commissions and the provision of credit. Established policies of MAP traders and middlemen at times work to the disadvantage of local collectors. Collectors often have an urgent need for cash. This provides a strong advantage to local shopkeepers and dealers, in that it at times allows them to purchase collectors’ MAPs products at a discount. Given that collectors are often cash poor, they are often willing to agree to lower prices in order to receive cash.Figure 3 also shows that opportunities exist to improve collector/farmers profit margins. Disadvantages can be eliminated through capacity building of collectors in pre and post-harvest handling, general cash management, and through micro-finance opportunities. Collectors/farmers are in dire need of training in order to understand how best to meet market needs, and to understand how value addition impacts product demand and ultimately sales. Improvements are needed in the collectors’ skills and abilities to produce marketable surpluses, market linkages (including the implementation of secure trade contracts), and the ability to store product (for example, a species collected now may bring about a higher price six months later). In this way, collectors may begin to market products more effectively, which could transfer a portion of the margin from middlemen to collectors. To enhance incomes from MAPs, collectors require a better understanding of the needs of individual markets. This is critically important, and especially regarding quality specifications, and their impact on pre and post-harvest management, and proper product handling.

Given that the harvesting of MAPs is a part-time activity, and the quantity of MAPs collected by individuals is relatively small, there is also an opportunity to market products via collector “clusters”. The need for an increase in direct marketing by collectors is becoming more and more important. The trade of most MAPs is highly competitive, with strong competition coming from China, India, and other neighboring countries. The ability to bypass certain middlemen would effectively shorten the value chain and would make products from Swat District more competitive in the market place (allowing Pakistan to compete more strongly with other providers), and again should increase collectors’ margins.

Potential also exists for the use of MAP products in new and emerging sector niches, including certain types of food, beverages, pharmaceuticals, and perfumes. Another potential sector is in the pesticide industry, and especially insecticides. Since there is an increasing awareness regarding adverse effects of chemical pesticides, products of natural origin (especially when collected from the wild) have received increasing interest of late.Figure 3 shows that lack of clear and specific government policies for MAPs is a serious challenge and root cause of difficulties as perceived by the MAPs collectors and dealers. Lack of government support and cultural restrictions, as well as a lack of clear and specified policy provisions in relation to collection, transport, and sale, are also contributing to low monetary return from MAPs business and collection. Many of the traders complained that the government’s policies in regulating MAPs were not realistic considering the current trends in the business. They also complained that the government was not giving sufficient support to these enterprises and trade. This may be due to increased awareness by traders of the threat posed by the declining availability of local raw materials and growing market competition. However, most respondent did not rate access to finance, business development services, markets, marketing information, and infrastructure as problematic. The need for business development services is still underestimated and there was little recognition of this issue and challenge.

### Prices along the marketing chain

Estimated prices along the marketing chain for the 24 reported high value MAPs sold in largest quantities by the surveyed collectors in Swat District are presented in Table 1. The prices reported in Table 1 were obtained through the questionnaires and focus group discussions with stakeholders. In the MAPs business price fluctuation is very high, and prices per species vary not only from year to year, but also frequently due to changes in demand and supply. Therefore, brochures/price list of the exporters and pharmaceutical companies were also used as a cross reference. The prices reported in Table 1 are our best assessment of averages for 2012.

**Table 1:** **High value MAPs of District Swat origin with their incremental values at different stages of the trade chain, 2012**

Price (Rs/Kg)											
Scientific name (vernacular name in (Pashto)	Family	VSN	Part sold	Quantity (Kg)	Collectors/farmers	Swat retail	Swat whole sale	National market	International market	Increase (international market vs. received by collectors/farmers)	Collector revenue (Rs)
*Aconitum heterophyllum* Wall. ex Royle (Zaharmora)	Ranunculaceae	UOS201	Rh	1,000	20	40	160	200	300	15.0	20,000
*Acorus calamus* L. (Skhawaja)	Acoraceae	UOS202	Rh	3,000	30	25	50	90	200	6.67	90,000
*Adiantum capillus-veneris* L. (Persosha)	Adiantaceae	UOS203	Wp	4,000	20	30	50	100	250	12.5	80,000
*Asparagus adscendens* Roxb. (Muslisufaid)	Liliaceae	UOS204	Rh	2,000	50	100	160	320	500	10.0	100,000
*Berberis vulgaris* L. (Kwaray)	Berberidaceae	UOS205	B	4,000	200	250	300	350	450	2.25	800,000
*Bergenia ciliata (Haw.) Sternb.* (Makanpath)	Saxifragaceae	UOS206	Rh	3,000	100	150	200	250	300	3.0	300,000
*Persicaria amplexicaulis* (D. Don) Ronse Decr. (syn. *Bistorta amplexicaulis* (D. Don) Greene) (Anjabar)	Polygonaceae	UOS207	Rh	12,000	60	100	150	200	300	5.0	720,000
*Bunium persicum* (Boiss.) B.(Tora Zera)	Apiaceae	UOS208	Fr	1,000	400	430	450	600	1,000	2.5	400,000
*Colchicum luteum* Baker (Suranjan)	Colchicaceae	UOS209	C	3,000	100	190	230	350	500	5.0	300,000
*Commiphora mukul (Hook. ex Stocks) Engl.* (Guggal)	Burseraceae	UOS210	Fl	5,000	100	150	220	350	650	6.5	500,000
*Dioscorea deltoidea Wall. ex Griseb.* (Kanis)	Dioscoreaceae	UOS211	Rh	3,000	100	220	250	300	400	4.0	300,000
*Diospyros lotus* L. (Tour amlok)	Ebenaceae	UOS212	Fr	90,000	50	90	150	200	300	6.0	4,500,000
*Geranium wallichianum D.Don ex Sweet* (Srazela)	Geraniaceae	UOS213	Rh	2,000	190	250	300	500	100	5.26	380,000
*Jurinea himalaica* R.R. Stewart (Sharrsham)	Asteraceae	UOS214	Rh	2,000	70	70	90	150	300	4.29	140,000
*Morchella esculenta* Fr. (Guji)	Morchellaceae	UOS215	Wp	5,000	10,000	12,000	15,000	20,000	30,000	3.0	50,000,000
*Paeonia emodi* Royle (Mamekh)	Paeoniaceae	UOS216	Rh	5,000	50	70	100	150	250	5.0	250,000
*Pistacia chinensis* subsp. *integerrima* (J.L.Stewart ex Brandis) Rech.f. (Kakar singay)	*Anacardiaceae*	UOS217	Pod	1,000	250	350	400	600	1,000	4.0	250,000
*Sinopodophyllum hexandrum* (Royle) T.S.Ying (syn. *Podophyllum hexandrum* Royle (Bankarri)	Podophyllaceae	UOS218	Rh	2,000	70	150	200	300	500	7.14	140,000
*Polygonatum multiflorum* (L.) All. (Noory alam)	Asparagaceae	UOS219	Rh	5,000	50	80	100	200	350	7.0	250,000
*Trachyspermum ammi (L.) Sprague* (Ajwoin)	Apiaceae	UOS220	Fr	1,500	300	200	250	400	550	1.83	450,000
*Trillium govanianum* Wall. ex D. Don (Matarjarrai)	Melianthaceae	UOS221	Fr	8,000	350	400	450	500	800	2.29	2,800,000
*Valeriana jatamansi* Jones (syn. *Valeriana wallichii* DC.) (Muskay bala)	Valerianaceae	UOS222	Rh	2,500	90	100	200	300	400	4.44	225,000
*Viola pilosa* Blume (syn. *Viola serpens* Wall. ex Ging.) (Banafsha)	Violaceae	UOS223	Fl	4,000	500	550	600	1,000	1,500	3.0	2,000,000
*Viola pilosa* Blume (syn. *Viola serpens* Wall. ex Ging.) (Banafsha)	Violaceae	UOS224	L+Fl	7,000	200	220	300	500	800	4.0	1,400,000
**Total or Average**				**176,000**	**556**	**677**	**848**	**1,163**	**1,775**	**5.4**	**66,395,000**

Source: Data collection by the study. Estimates are based on surveys and interviews with collectors, dealers and hakims and other information consulted. These are our best estimates for 2012 but may not be very accurate.

Note: Voucher Specimen Number (*VSN*): Roots (*Rh*); Whole plant *(Wp*); Fruits (*Fr*); Bark (*B*); Pod (*Pod*); Flower (*Fl*); Leaf (*L*).

The prices of the high value MAPs increase at each step in the supply chain, as Table 1 shows. This increase constitutes two main factors: 1) incremental transportation and labor costs; 2) profit in support of the involved individuals. Another factor contributing to the price increase is that some plant material is lost at each level from such processing activities as cleaning, processing, grading, and packing etc. This weight loss varies from species to species and the modes of processing for sale. A fourth factor affecting the increase in price from the collector to the final point of sale in Pakistan are the collectors’ lack of knowledge concerning appropriate procedures for preparing the plant material in such a way that it maintains the maximum possible value, as well as their general ignorance concerning prevailing prices and demand, as discussed in relation to Figure 3.

The MAPs species that on average brought collectors by far the highest price in 2012 was *M. esculenta* (10,000 Rs/kg). This was followed at lower levels by *V. pilosa* (flowers only, 500 Rs/kg) and *Bunium persicum* (Boiss.) B. Fedtsch. (400 Rs/kg). The greatest increase shown in Table 1 in both the national and international price from collectors to consumers was for *Aconitum heterophyllum* Wall. ex Royle (10 times higher in the national market and 15 times higher in the international market compared to the purchase price of collectors in Swat District). Other large price differentials are for *Asparagus adscendens* Roxb. (6.4, 10) and *Adiantum capillus-veneris* L. (5, 12.5). As is shown by the two rows for *V. pilosa*, the value of the plant material is determined by what is in the sample, not just the species involved. Flowers of *V. pilosa* sell for 500 Rs/kg at the collector level, a mixture of leaves and flowers for 200 Rs/kg. For these products, the international price is 3–4 times the price received by collectors. The price differentials for other species can similarly be traced in Table 1.

### Quantities collected

Our surveys of collectors and dealers generated estimates of the quantities of the 24 high value MAPs marketed from Swat District by dealers and traders as well as prices at different marketing levels. The estimated quantities delivered to markets by the 120 surveyed collectors are shown in Table 2 per sub-valley and are summed up in Tables 1 and 2.

**Table 2:** **MAP quantities take out from different sub-valleys of District Swat during 2012**

Scientific name (vernacular name in (Pashto)	VSN	Transported outside valleys (Kg)						Total (Kg)
		Miandam	Madyan	Behrain	Kalam	Sulatand	Lalku	
*Aconitum heterophyllum* Wall. ex Royle (Zaharmora)	UOS201	250	205	95	350	45	55	1,000
*Acorus calamus* L. (Skhawaja)	UOS202	595	750	400	455	500	300	3,000
*Adiantum capillus-veneris* L. (Persosha)	UOS203	1500	1450	290	305	275	180	4,000
*Asparagus adscendens* Roxb. (Muslisufaid)	UOS204	560	680	345	120	105	190	2,000
*Berberis vulgaris* L. (Kwaray)	UOS205	1060	1050	235	145	665	845	4,000
*Bergenia ciliata* (Haw.) Sternb. (Makanpath)	UOS206	765	610	385	440	345	455	3,000
*Persicaria amplexicaulis* (D. Don) Ronse Decr. (syn. *Bistorta amplexicaulis* (D. Don) Greene) (Anjabar)	UOS207	2500	3550	2030	1550	1410	960	12,000
*Bunium persicum* (Boiss.) B.(Tora Zera)	UOS208	205	200	180	280	60	75	1,000
*Colchicum luteum* Baker (Suranjan)	UOS209	70	950	800	1030	75	75	3,000
*Commiphora mukul* (Hook. ex Stocks) Engl. (Guggal)	UOS210	1700	1350	600	525	332	493	5,000
*Dioscorea deltoidea* Wall. ex Griseb. (Kanis)	UOS211	500	845	355	500	450	350	3,000
*Diospyros lotus* L. (Tour amlok)	UOS212	55,000	20,000	10,000	1,000	3,500	500	90,000
*Geranium wallichianum* D.Don ex Sweet (Srazela)	UOS213	500	740	300	165	95	200	2,000
*Jurinea himalaica* R.R. Stewart (Sharrsham)	UOS214	560	475	360	215	290	100	2,000
*Morchella esculenta* Fr. (Guji)	UOS215	1,300	1,200	1,000	500	450	550	5,000
*Paeonia emodi* Royle (Mamekh)	UOS216	950	1250	850	700	600	650	5,000
*Pistacia chinensis* subsp. *integerrima* (J.L.Stewart ex Brandis) Rech.f. (Kakar singay)	UOS217	350	200	55	45	150	200	1,000
*Sinopodophyllum hexandrum* (Royle) T.S.Ying (syn. *Podophyllum hexandrum* Royle (Bankarri)	UOS218	460	600	500	180	165	95	2,000
*Polygonatum multiflorum* (L.) All. (Noory alam)	UOS219	1095	705	1150	1050	400	600	5,000
*Trachyspermum ammi* (L.) Sprague (Ajwoin)	UOS220	350	250	300	400	100	150	1,500
*Trillium govanianum* Wall. ex D. Don (Matarjarrai)	UOS221	2,500	1,500	1,000	2,000	400	600	8,000
*Valeriana jatamansi* Jones (syn. *Valeriana wallichii* DC.) (Muskay bala)	UOS222	800	900	400	195	100	105	2,500
*Viola pilosa* Blume (syn. *Viola serpens* Wall. ex Ging.) (Banafsha)	UOS223	1,500	1,200	500	450	150	200	4,000
*Viola pilosa* Blume (syn. *Viola serpens* Wall. ex Ging.) (Banafsha)	UOS224	2,000	2,500	1,000	500	600	400	7,000

*VSN*, voucher specimen number.

The quantities indicate that *Diospyrus lotus* L. (90,000 kg) was collected in by far the largest quantity, followed by *Persicaria amplexicaulis* (D. Don) Ronse Decr. (syn. *Bistorta amplexicaulis* D.Don) (12,000 kg) and *V. pilosa* (11,000 kg, combined flowers and leaves and flowers). *Trillium govanianum* Wall. ex D.Don (8000 kg), *M. esculenta, Paeonia emodi* Royle and *Polygonatum multiflorum* (L.) All. (each 5000 kg), and *A. capillus-veneris* and *Berberis vulgaris* L. (each 4000 kg) were also collected in reasonably great quantities from the surveyed areas and sold in the herbal markets of Mingora and Madyan. Other species were collected in quantities of 3000 kg or less from the studied sites of Swat District.

The present assessed quantities taken out of Swat District are not based on a managed optimum exploitation of these species in the valleys. Market demands for the most of these species may exceed the existing supply, as the interviews with collectors/farmers suggested larger amounts of these species could have been sold. Similarly, the interviews suggested potential supply can be higher than the present amounts taken out. The differences are due to uncoordinated demand and supply and unawareness about the availability of certain species and their demand in the market. However, sustainability of harvests of endangered MAP species also has to be taken into account as a long run concern.

### Estimated income of participants in MAP trade

Different stakeholders of the MAP business, e.g. collectors/farmers, agents, shopkeepers, wholesalers, representatives of pharmaceutical companies, were asked about their annual income during the market study. The respondents were reluctant to disclose their income from the MAP business and the responses were invariably discouraging. The majority of them declined to answer this question. The responses from a few people were not in agreement with the quantities and price of materials they claim to sell annually. The only reliable information which came out of this survey from the direct questions regarding the income of different players in the MAP trade was very general. It was observed that the collectors of crude material were invariably on the losing side, earning comparatively little from their hard work. On the other hand, middlemen, wholesalers, and retailers earn larger incomes from this trade.

More specifically, collectors/farmers who rent their lands/forest areas were hesitant to give details about their income because they did not wish this information to be passed on to the landowners. The traders were also reluctant to give details due to a fear that the information would be passed to the tax authorities. However, our study revealed that the trade of MAPs is dominated by a few wholesalers and hakims in the surveyed areas. Overall, it was generally observed in the interviews with local collectors, farmers, and dealers, that in the surveyed sub-valleys, the local middlemen receive the most handsome returns. It was also observed that the local wholesalers control price information to the collectors, and that enables them to maintain high profits.

Some estimate of the total income earned at the collector level can be derived from the price and quantity estimates in Table 1. Based on the estimated quantities taken out from the six sub-valleys of Swat District in 2012, and the estimated prices obtained at the collector level, the total revenues were about Rs. 50 million from *M. esculenta* and Rs. 16.4 million from the other 23 species. Among these 23 species, the MAPs that were the best income source for the collectors and gatherers, because of their combination of price and quantity, were *D. lotus, T. govanianum* and *V. pilosa*. The price for *B. persicum* (400 Rs/kg) was third highest, but the amount sold was relatively low. The total collector revenue generated from the sale of the 24 MAPs by the 120 surveyed collectors in Swat District is about Rs. 66.4 million.

Some additional illustrative income calculations can also be based on the prices and quantities shown in Table 1. The total quantity of the 24 high value MAPs estimated to have been collected is 176,000 kg. This averages 1,467 kg per collector for the 120 interviewed in the survey. The estimated total revenue at the collector level of Rs. 66.4 million implies an average of Rs. 553,291 per collector, again based on the surveyed 120 collectors involved in marketing the quantities of MAPs indicated in Table 1. With a large proportion of the income coming from just one species and collection of that species always uncertain, there is substantial variability of income around the estimated average.

If the further assumption is made that about 5,000 nomadic tribesman provide the MAPs brought to market by the 120 surveyed collectors, the average quantity supplied is 35.3 kg per gatherer household. The average revenue of a gatherer household is Rs. 13,279.

While our estimate of collector revenue is higher than some earlier estimates [18, 28, 29], there are also reasons to believe it still understates incomes from MAPs collection. In our survey of collectors and focus group data collection, individual respondents reported that the amount of MAPs gathered per household per year was between 12 kg and 150 kg. Using an average of 80 kg, and again assuming 5,000 gatherers are providing the MAPs marketed by the 120 collectors, this would imply quantities of about 400,000 kg of MAPs collected (80 kg per gatherer household × 5,000 households). This estimate is more than double the quantity reported in Tables 1 and 2 based on collector and dealer interview responses for quantities of the specific MAPs. While this larger quantity would include the other 56 MAPs collected in addition to the 24 high value MAPs identified in the tables, the collection of these additional species may not fully explain the difference in quantity estimates. Collection of higher quantities than shown in Table 1 would increase the total and average incomes estimated from MAP collection.

One check on these illustrative calculations comes from estimating an implied daily wage for MAP gatherers. Our study suggests that most income from MAPs is earned during a four-month spring/summer period of collection. The interviews suggest that an average of about 1.7 persons are engaged in gathering and collection per household. Assuming they work six days per week during this four-month period, each household would have spent about 174 person days on MAPs. The total employment under the assumption of 5,000 gatherer households would be 870,000 person days providing the MAPs marketed by the surveyed collectors. Using the revenue estimates from Table 1 and this estimate of level of employment, an average daily earnings per person from gathering MAPs can be computed of Rs. 76.3 per day (Rs. 13,279/174 person days of work). This estimate of daily earnings is somewhat lower than observed daily wages for unskilled agricultural workers in Swat District. The wage earned from MAPs collection would be closer to competitive labor wages if larger quantities are actually collected or fewer gatherers are providing these quantities. But the average estimated daily earning is plausible for several reasons. MAPs are mostly gathered by nomads as a part-time activity/business, and their working/ laboring skill are lower than the unskilled agricultural labor. Secondly, they spend only 4–5 hours per day gathering MAPs within a 2–3 km distance. In 2012, daily wages for unskilled agricultural labor was about Rs. 250 per day (Rs. 200 in cash and a lunch meal valued at about Rs. 50) for eight hours of work. The 4–5 hours per day is 50 percent less investment of work of the MAPs gatherers than the unskilled agricultural workers. On this basis, the estimated revenue per gatherer household from the calculations above are reasonable.

### International trade of MAPs

Pakistan both exports and imports substantial quantities of herbal material in trade with other countries. The bulk of the MAPs materials are exported from developing countries while major markets are in the developed countries [27]. In 2012, Pakistan as a whole exported such plant materials worth over US $10.5 million [30]. Data is only available in the form of condiments and under title of other spices all the MAPs are summed up. Their export and import values are listed in the Foreign Trade Statistics of Pakistan. The herbal markets of Karachi (Jodia Bazar) and Lahore (Akbari Mandi) act as a main source for MAPs export. The reported destinations of exports include Germany, USA, the Middle East, Switzerland and many other countries. The share of Swat District in the export market is estimated to be substantial, possibly as high as 40% or more [15, 31, 32].

Similarly in 2012, the import of herbal material as a whole was worth over US$ 130 million annually [30]. The herbal market of Lahore, Akbari Mandi, acts as the main hub and receives very large quantities of imported herbs from India and more recently China. Other sources of imports include Thailand, Indonesia, Tanzania, Iran, and Afghanistan. An increasing market trend of imports has occurred, particularly from India, China, Iran and Afghanistan. This is partly attributed to an increasing demand in domestic markets of Pakistan. It is also attributed by respondents in our study to inferior quality of indigenous raw material and uncertainty about the timing of delivery to market. In this context, proper education and awareness of the collectors/farmers and dealers will not only save foreign exchange but will also open new avenues for low income groups, therefore, playing a role in poverty alleviation. It is important to note that in Pakistan the foreign trade through unconventional routes, including cross border exchanges, is often unmonitored and is part of the undocumented economy of the country.

## Discussion

The geographical location of Swat District provides an ideal physical environment for the growth and nourishment of many high value medicinal and aromatic plants. These crops can make a contribution to the economic development of the area in particular and the country in general. This study of the trade patterns of MAPs from Swat District was based on surveys, interviews, and focus group meetings with participants in the market value chain including collectors/farmers, local dealers, shopkeepers and hakims, wholesalers, representatives of the domestic pharmaceutical industry, and exporters. The study reveals that various species from Swat District are sold in large quantities both in national and international markets, indicating their importance as a source of income for the nomadic tribesman and small farmer inhabitants of the mountain communities. At the same time few species harvested in Swat District have been reported from market abroad, but this could be due to the difficulty of species identification in trade or the lack of endemics that can be used as place-holders [33]. The study generally observed that the city of Mingora is the collection and trading center for many high value MAPs, having a well-established market that supplies various trading centers in Pakistan and abroad. Rashid et al. [31] also reported that the majority of marketable medicinal plants are collected from northern areas of Pakistan including Swat District.

The MAPs trade in Pakistan, including Swat District, operates with minimal state intervention and documentation. This informal market is present in many countries and provides an important source of income to both subsistence farmers, traders, middlemen, vendors, wholesalers and retailers [11–13, 22, 26, 34–40]. Because of this market operates in grey zone, decision makers are usually unaware of the significance of the trade in MAPs and of the negative impacts that unsustainable harvesting of these plants may have on the environment and on people’s long term welfare. The concerns of overharvesting have been raised by many authors that have found reducing availability of plant species or through informant reports [41–44]. A vibrant private market is a desirable outcome as long as none of the participants are able to exploit those at a lower level in the marketing chain and as long as the natural environment is not deteriorated by the over-harvesting of the collectors. These concerns can justify educational efforts, collective marketing activities by collectors, and regulations of harvesting as government policies.

Pakistan is a signatory of the Convention on Biological Diversity (CBD), the Convention on International Trade in Endangered Species of Wild Fauna and Flora (CITES), and various others [45]. Hence Pakistan has recognized the importance of value and conservation of its biological diversity. The Ministry of Environment has drafted a Biodiversity Action Plan (BAP) in collaboration with involved stakeholders. BAP has proposed actions for *in-situ* and *ex-situ* conservation of Pakistan’s biodiversity including MAPs. However, genetic diversity of traditional MAPs is continuously under the threat of extinction due to unsustainable harvesting techniques. A balance is needed between the value of MAPs for the products derived from them and their environmental preservation. Maintaining the supply of MAPs is a problem mainly because most of them are harvested from the wild. As the trade has become market-oriented and international, the activities of a growing number of gatherers are outstripping natural MAP populations. Hence, there is need to establish this trade on a more scientific and sustainable basis. The vulnerability of MAPs to over-exploitation and threat of extinction needs to be dealt with pragmatically. However, the country has still to go a long way to frame laws and policies to conserve all its genetic materials and to develop bilateral and multilateral exchange of plant germplasm, appropriate breeders, farmers and community rights, and comprehensive action plans to achieve the objectives of trade sustainability and its linkages with conservation.

There is no systematic management structure involved in collection of MAPs plant material in Swat District, which appears to be on a first-come basis. This is cause for concern because, as Balick [46] and Saganuwan [47] reported, the lack of any check, even on the collection of rare or threatened species, endangers this important source of income. Similarly, Larsen and Smith [48] examined stakeholder perspectives on commercial medicinal plant collection in Nepal, and noticed that most of the commercially important MAPs were becoming rare and sparse due to the combination of unregulated collection and overgrazing.

In the interviews for this study, very few of the collectors knew about the existence of the major markets for MAPs or were sufficiently trained in such critical skills as the best pre and post-harvest treatments. Olsen and Larsen [49] also found that the trade and collection of plant materials is mostly practiced by unskilled persons. As a result, valuable medicinal plants lose value on their way to their final market while being subject to over-extraction and destructive harvesting techniques. This suggests that there is a need to develop practical and economically sound strategies for the efficient utilization natural resources in order to improve the condition of marginalized communities; a process that should eventually lead the country towards greater economic stability.

Household incomes of gatherers and collectors of MAPs can be enhanced if communities acquire a better understanding of the economic importance of high-demand natural plant resources, as well the interests and respective roles of the key stakeholders involved in the market. This must include understanding of the importance of sustainable collection and cultivation practices to the long term livelihood of the community. Educational programs should address the issue of sustainable harvesting and the possibility of cultivating MAPs that are currently collected from the wild. There is also need for a program that focuses on assisting collectors and local dealers in providing consistently high quality, well preserved, material to purchasers, combined with a sharing of information as to why this is important. This paper provides a baseline analysis of the marketing channels and price differences that underscore these needs.

The study provides estimates of the price increases as high value MAPs move through the supply chain. These price differences are to be expected, but are also exacerbated by the lack of knowledge among collectors and local dealers concerning the demand for the various plants. Nomadic gatherers and collectors usually have no direct knowledge of the final markets for their products and, therefore, must rely on local traders to sell their products within the existing value chain. To enhance their income from MAPs, collectors and local dealers need a better understanding of the markets they are supplying. This means understanding the extent and seasonality of the demand and market prices as well as quality expectations and their impact on appropriate pre-harvest and post-harvest management and handling of MAPs species.

The perceived threats to plant conservation and sustainability of medicinal plant harvesting do require further study to ascertain that these threats are applicable to the situation in Swat District. Larsen and Olsen [50] argue that standard assumptions on medicinal plant trade can be flawed, such as the degradation of the commercial medicinal plant resource base, the open-access of medicinal plant source areas, the contribution of commercially collected medicinal plant species to conservation, and that medicinal plant harvesters are cheated by middlemen. The data presented here supports the assumption that middlemen and especially harvesters profit the least among the market chain, but other aspects should be studied in the field in multi-year study to assess the changes over time.

## Conclusion

The purpose of this study was to examine the current status of the high value medicinal and aromatic plant trade in Pakistan and investigate the linkages in the market chain from collectors in Swat District to final consumers. During the course of the interviews and focus group meetings of the study, a range of possible interventions were identified that would enable MAPs to become a stronger ‘engine of growth’ for the local economy. Indeed, simply conducting the study helped inform local collectors/farmers about the value-added products derived from MAPs. It is reasonable to expect that improving market linkages between producers and buyers will result in increased economic benefit for local collectors, farmers, and dealers, enabling their communities to become hubs of significant economic activities with a multi-dimensional impact on the economic development of Swat District. Such developments are also essential if Pakistan is to maintain or improve its position as an international supplier of MAPs.
